# A Simple Remedy for Thrush and Sordes

**Published:** 1890-03

**Authors:** 


### A Simple Remedy for Thrush and Sordes.

Among the ill-fed children of the poorest residents of our large cities, thrush is an extremely common and troublesome complaint. The following lotion, to be applied frequently with a feather or brush to the white patches, kills the oidium albicans more quickly than any other I know, and removes the patches after a few applications, leaving healthy mucous membrane. It consists of equal parts of lotio nigra and glycerine mixed. I attribute its action to the germicidal power of the mercury. The quantity used is so small as to be quite harmless. Another condition in which I have found the same lotion invaluable is in that of the sordes which collect so abundantly on the teeth, lips and tongue in many cases of enteric fever. It cleans these parts as if by magic, and renders that unpleasant process known as "scraping the tongue" quite unnecessary. It may also with advantage be painted over the fauces, etc., in those unhealthy conditions of the throat which are so common in typhoid. I tried it in one case of catarrhal stomatitis, but it had no effect, whereas chlorate of potash effected an immediate cure. Also, in the sordes of advanced phthisis it seems to be of no use. Not having seen this lotion mentioned in any book, and having found it superior to any of the usual preparations in use for these affections, I venture to bring it to the notice of the profession, in the hope that it may prove as great a boon to other practitioners as it has been to myself.—Ord, *The Lancet.*

---

The Postal Laws make it larceny to take a newspaper and refuse to pay for it. A newspaper in Illinois recently brought suit against forty-three men who would not pay their subscriptions, and obtained judgment in each for the full amount of the claim. Of these, twenty-eight men made affidavit that they owned no more property than the law allowed them, thus preventing attachments. Then they, under the decision of the Supreme Court, were arrested for petty larceny, and bound over in the sum of $300 each. All but six gave bond, while six went to jail.—*Pharm. Record.*

